# Cross-Tissue Characterization of Heterogeneities of Mesenchymal Stem Cells and Their Differentiation Potentials

**DOI:** 10.3389/fcell.2021.781021

**Published:** 2021-12-17

**Authors:** Wenhong Hou, Li Duan, Changyuan Huang, Xingfu Li, Xiao Xu, Pengfei Qin, Ni Hong, Daping Wang, Wenfei Jin

**Affiliations:** ^1^ Shenzhen Key Laboratory of Gene Regulation and Systems Biology, School of Life Sciences, Southern University of Science and Technology, Shenzhen, China; ^2^ Department of Orthopedics, Shenzhen Second People’s Hospital, The First Affiliated Hospital of Shenzhen University Health Science Center, Shenzhen, China; ^3^ Shenzhen Institute of Geriatircs, Shenzhen, China; ^4^ School of Life Science and Technology, Harbin Institute of Technology, Harbin, China

**Keywords:** Mesenchymal stem cells, single cell RNA-seq, cellular heterogeneity, cellular differentiation, chondrogenic differentiation, adipogenic differentiation, osteogenic differentiation

## Abstract

Mesenchymal stem/stromal cells (MSCs) are promising cell sources for regenerative medicine and the treatment of autoimmune disorders. Comparing MSCs from different tissues at the single-cell level is fundamental for optimizing clinical applications. Here we analyzed single-cell RNA-seq data of MSCs from four tissues, namely umbilical cord, bone marrow, synovial tissue, and adipose tissue. We identified three major cell subpopulations, namely osteo-MSCs, chondro-MSCs, and adipo/myo-MSCs, across all MSC samples. MSCs from the umbilical cord exhibited the highest immunosuppression, potentially indicating it is the best immune modulator for autoimmune diseases. MSC subpopulations, with different subtypes and tissue sources, showed pronounced differences in differentiation potentials. After we compared the cell subpopulations and cell status pre-and-post chondrogenesis induction, osteogenesis induction, and adipogenesis induction, respectively, we found MSC subpopulations expanded and differentiated when their subtypes consist with induction directions, while the other subpopulations shrank. We identified the genes and transcription factors underlying each induction at the single-cell level and subpopulation level, providing better targets for improving induction efficiency.

## Introduction

Mesenchymal stem/stromal cells (MSCs) are multipotent stromal cells that can differentiate into a variety of cell types including chondrocytes, osteocytes, myocytes, and adipocytes *in vivo* and *in vitro* (Pittenger et al., 1999; Dominici et al., 2006; Salem and Thiemermann, 2010). MSCs also played an important role in tissue homeostasis and immunomodulation *via* interaction with immune cells and secretion of various factors including growth factors, cytokines, and antifibrotics (Ren et al., 2008; Djouad et al., 2009; Nemeth et al., 2010). Due to MSCs showing exciting features such as self-renewal capacity, directional differentiation, and immunomodulation, they have become the most promising cell source for cell-based therapies, particularly in tissue repair and treatment of immune disorders (Krampera et al., 2006; Ren et al., 2008; Djouad et al., 2009; Salem and Thiemermann, 2010; Hare et al., 2012). In particular, the delivery of Human MSCs to injured tissues has shown several important functions of MSCs, namely immunomodulation, reduction of fibrosis, stimulation of neovascularization, and endogenous tissue regeneration, work in parallel (Djouad et al., 2009; Hare et al., 2012) The number of clinical trials on MSCs-based therapies is rising considerably in recent years, further indicating the great potential of MSCs. However, dysregulation of MSCs induction and low efficiency of MSCs induction into target functional cells remain hinders the application of MSC-based therapies.

Although there are established approaches for induction of MSCs into specific functional cell types *in vitro* (Colter et al., 2000; Muraglia et al., 2000; Lee et al., 2016), Most studies showed that MSCs exhibited significant differences in colony morphologies, proliferation rates and differentiation potentials (Colter et al., 2000; Muraglia et al., 2000). The lack of a clear understanding of the cellular heterogeneity of MSCs severely hampered the development of an efficient and reproducible clinical application (Yoo et al., 2009). Furthermore, MSCs have been identified in most tissues in our body and could be isolated from bone marrow, umbilical cord, adipose tissue, synovial tissue, muscle, liver, dental pulp, and so on Prockop (1997), Zuk et al. (2002), Romanov et al. (2003), Wang et al. (2004). It remains largely unexplored whether MSC subpopulations are consistent across tissues. In particular, the response heterogeneity of MSCs from different tissues to inductions is unknown. Furthermore, our knowledge on lineage commitment of MSCs is mainly based on analysis of bulk assay, which captures the average signal across entire populations while ignored response heterogeneity of MSCs. Investigating the response of MSCs to various inductions at single-cell resolution could greatly facilitate our understanding of the processes and aid in developing efficient induction approaches.

Although single-cell RNA-seq (scRNA-seq) is very powerful to reveal cellular heterogeneities under various conditions (Macosko et al., 2015; Wang et al., 2019; Qin et al., 2021), there are only a few studies that explored the heterogeneities of MSCs with a limited number of cells or limited samples (Huang et al., 2019; Merrick et al., 2019; Rauch et al., 2019). Here we analyzed scRNA-seq data of MSCs from multiple tissues to elucidate MSC subpopulation across tissues. We found the MSC subpopulations from different tissues were essentially consistent with each other, although the abundance of each subpopulation was highly diverse across tissues. Induction of MSCs into different functional cell types was used to provide novel insight on mechanisms of MSCs differentiation. We further identified transcription factors underlying each induction at single-cell level to elucidate the potential target for efficient induction. Our work serves as a valuable resource for the MSCs community and could improve our understanding of the basic functional characteristics of MSCs and enhance the induction efficiency of specific MSC lineage.

## Results

### scRNA-Seq Showed Consistent MSC Subpopulations Across Tissues

**FIGURE 1:**
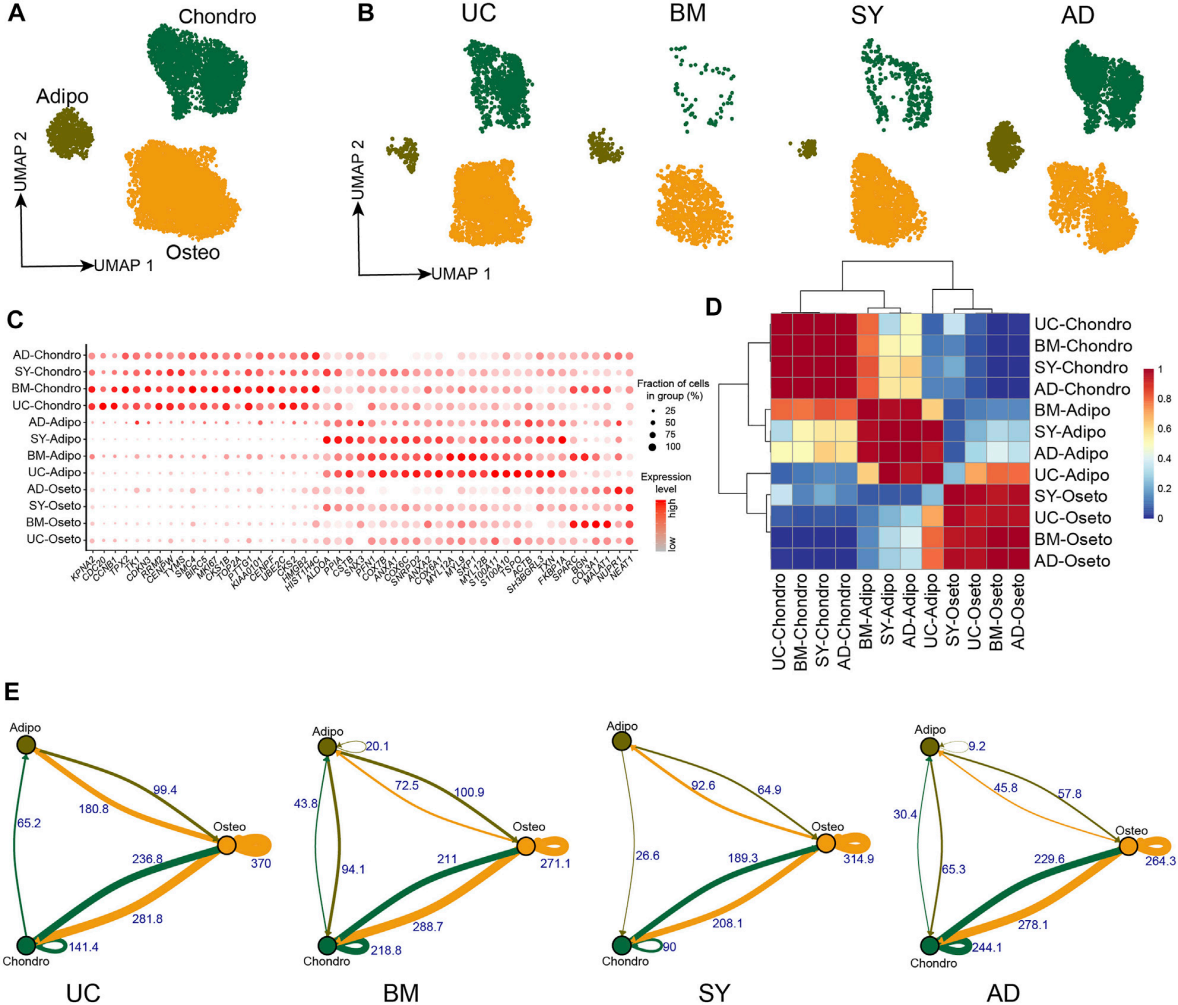
MSC subpopulations in 4 different tissues. **(A)**. UMAP projection of MSCs in 4 different tissues, colored by MSC subpopulations. **(B)**. UMAP projection of MSCs from each tissue, namely UC-MSCs, BM-MSCs, SY-MSCs, and AD-MSCs. **(C)**. Dot plot of the expression of subpopulation-specific genes in the MSC subpopulations. **(D)**. Heatmap of AUROC scores between MSC subpopulations pairs, in which AUROC scores represent similarities between subpopulations. **(E)**. Weighted ligand-receptor interactions between MSC subpopulations pairs in each tissue based on STRING database. The edge thickness and corresponding numbers indicate the sum of weighted paths between subpopulations.

Human umbilical cords (UC) and synovial tissue (SY) were collected from Shenzhen Second People’s Hospital, with donors signing their informed consent approved by the IRB of the hospital. UC-MSCs were isolated from UC Wharton’s jelly following Reppel et al. (2014); and SY-MSCs were isolated from the synovial membrane of knee joints following De Bari et al. (2001). The scRNA-seq data of UC-MSCs and SY-MSCs were generated following 10X genomics protocol. The scRNA-seq data of MSCs from bone marrow (BM) (Rauch et al., 2019) and adipocytes (AD) (Liu et al., 2019) were integrated for analyses of the cellular heterogeneities of MSCs across different tissues. Unsupervised clustering of each of the four MSCs samples resulted in three distinct clusters, respectively (Supplementary Figures S1A–H and Supplementary Table S1). As expected, we identified the 3 major cell subpopulations on Uniform Manifold Approximation and Projection (UMAP) projection after we integrated the four MSC samples (Figure 1A). All cell subpopulations from the four tissues expressed MSCs specific markers such as *THY1* (CD90), *NT5E* (CD73), and *ENG* (CD105) (Supplementary Figure S1I). Each MSC sample has the three subpopulations but with different abundances (Figure 1B), indicating the MSC subpopulations are essentially consistent among these MSC samples. The three MSC subpopulations exhibited lineage-specific expression, namely chondrocyte lineage (*HMGB1*, *HMGB2, DCN, F3*, *MDK, BMP5, KIAA0101*), adipocyte/myocyte lineage (*FTL*, *FTH1*, *TAGLN, FKBP1A*, *ACTG2*, *TXN*) and osteoblast lineage (*BGN*, *HAPLN1*, *FHL1*, *VCAN*, *GDF15*) (Figure 1C, Supplementary Figures S1B, S1D, S1F, S1H and Supplementary Table S2). We named the three subpopulations as chondrocyte lineage MSCs (chondro), adipocyte/myocyte lineage MSCs (adipo), and osteocyte lineage MSCs (osteo). MSCs deriving from different tissues while clustering into the same subpopulation exhibited similar lineage-specific expression profiles (Figures 1C,D), further indicating all the four MSC samples had the same cell subpopulations.

The crosstalk of ligand-receptor on the cell surface plays an important role in cellular signaling transduction. We analyzed the crosstalk of ligand-receptor pairs to understand the cell-cell communication in MSCs. We found osteo→osteo and osteo→chondro showed the strongest interaction among all subpopulation pairs in each MSC sample (Figure 1E, Supplementary Figures S2A–D). Therefore, oseto is the most important signaling sender in MSCs, potentially indicating osteo shapes and contributes to the cellular micro-environment. The crosstalk between osteo and chondro is the second strongest, potentially indicating there is frequent cell-cell communication between them. The crosstalk between adipo and osteo/chondro is very weak, potentially indicating that adipo are relatively isolated in MSCs. The observations are consistent with recent reports that adipogenesis and osteogenesis/chondrogenesis are mutually exclusive processes (Wolock et al., 2019a; Rauch et al., 2019).

### MSC Subpopulations From Different Tissues Show Different Potentials

**FIGURE 2:**
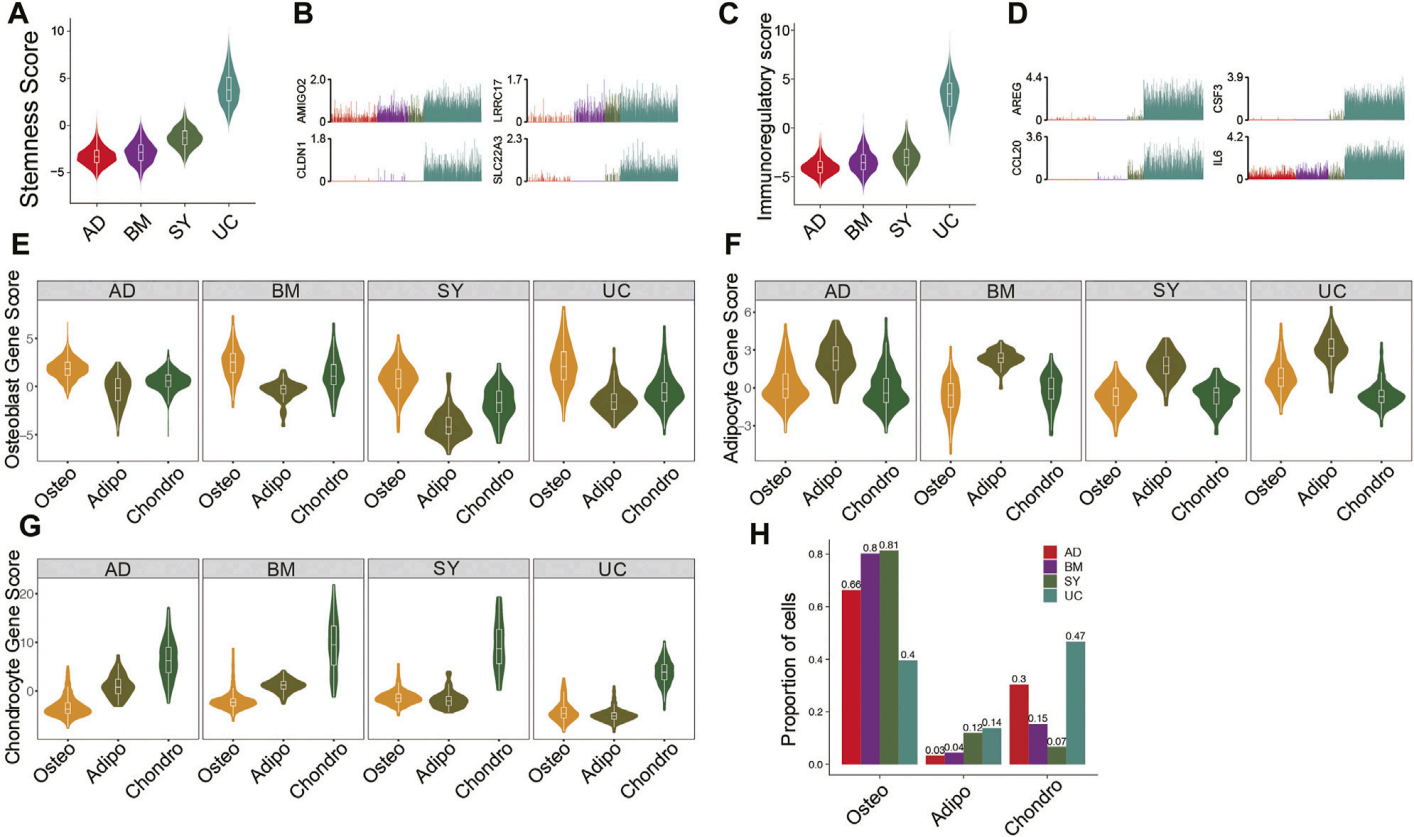
Features and lineage-specific differentiation potentials of MSC subpopulations. **(A)**. Violin plot of stemness scores of MSCs in 4 different tissues. **(B)**. Bar plot of the expression level of *AMIGO2, CLDN1, LRRC17,* and *SLC22A3* in each cell. **(C)**. Violin plot of immunoregulatory scores of MSCs in 4 different tissues. **(D)**. Bar plot of the expression level of *AREG, CSF3, CCL20,* and *IL6* in each cell. **(E–G)**. Violin plot of osteocyte score. **(E)**, adipocyte score. **(F)**, and chondrocyte score. **(G)** of MSCs in MSC subpopulations. **(H)**. Fractions of MSC subpopulations in MSCs for each tissue.

It is essential to compare the differentiation potentials and immunomodulatory ability of MSC subpopulations from different tissue sources due to their implications in MSC-based therapy. UC-MSCs always exhibited the highest stemness among all MSC samples (Figure 2A), potentially indicating their strongest proliferation potential and strongest stemness. Indeed, stemness-associated genes including *AMIGO2*, *CLDN1*, *LRRC17,* and *SLC22A3* were significantly highly expressed in UC-MSCs (Figure 2B). Furthermore, the violin plot showed UC-MSCs have the highest immunoregulatory scores among all MSC samples (Figure 2C), e.g., *AREG*, *CSF3*, *CCL20,* and *IL6* were significantly highly expressed in UC-MSCs (Figure 2D). These results potentially indicate that UC-MSCs are the best MSC source for immunomodulation of innate and adaptive immune responses, consistent with the reports based on bulk data (Meng et al., 2012).

Investigation of differentiation potential of MSCs to osteocyte lineage, chondrocyte lineage, and adipocyte/myocyte lineage are also crucial for clinical applications. The osteo subpopulations from the four tissues exhibited the highest differentiation potential to osteocytes, among which BM-osteo and UC-osteo had the highest osteocyte scores (Figure 2E). On the other hand, SY-adipo exhibited the lowest differentiation potential to osteocytes (Figure 2E), indicating it had to overcome much higher barriers to differentiate from osteocytes. Similar analyses showed that AD-adipo and BM-chondro had the highest differential potential to adipocyte lineage and to chondrocyte lineage, respectively (Figures 2F,G). Analyses of the abundances of MSC subpopulations from different tissues showed BM and SY have the highest fractions of osteo, potentially because both tissues are osteo associated (Figure 2H).

### Heterogeneous Response of MSCs to Chondrogenesis Induction

**FIGURE 3:**
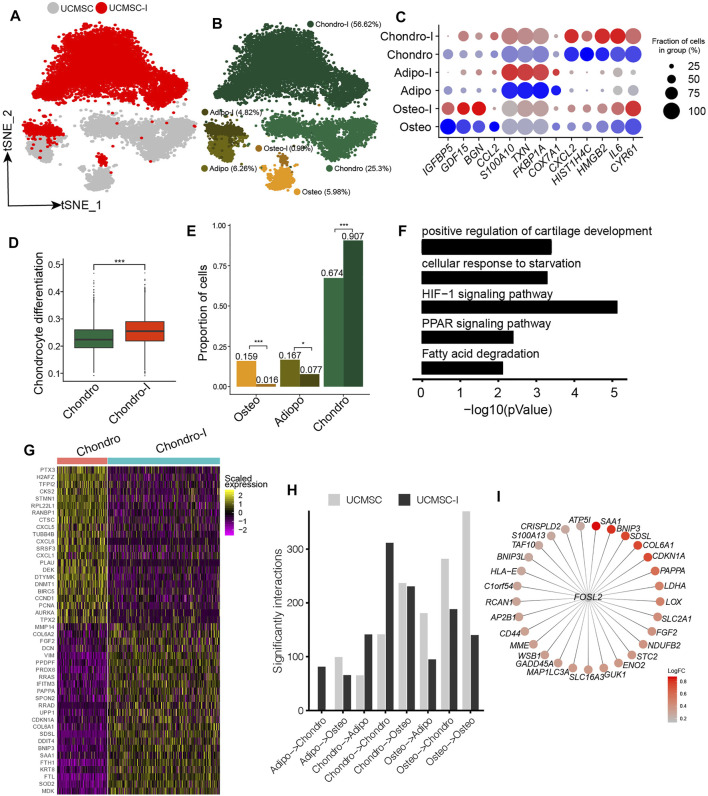
Response heterogeneity and dynamics of MSCs to chondrogenesis induction and its underlying mechanisms. **(A)**. t-SNE projection of MSCs pre-and post- chondrogenesis induction, colored by non-induced UC-MSC (UC-MSCs) and chondrogenic-induced MSCs (UCMSC-I). **(B)**. t-SNE projection of MSCs pre-and post- chondrogenesis induction, colored by the six MSC subpopulations, namely chondro, osteo, adipo, chondro-I, osteo-I, and adipo-I. **(C)**. Dot plot of the expression level of lineage-specific genes in MSC subpopulations pre-and post-chondrogenesis induction. **(D)**. Boxplot of chondrocyte differentiation scores of pre-induced chondro (Chondro) and chondrogenic-induced chondro (Chondro-I). Wilcoxon Rank Sum test, ***p < 0.001. **(E)**. Fractions of MSC subpopulation in MSCs and chondrogenic-induced MSCs. Fisher’s exact test, *p value <0.01, ***p value <0.001. **(F)**. The significantly enriched GO terms in chondro-I specific genes compared to non-induced chondro. **(G)**. Heatmap of the most significantly differentially expressed genes between pro-and post-induced chondro. **(H)**. Bar plot of significant interactions between MSC subpopulations pairs in UC-MSCs and UCMSC-I. **(I)**. Bubble plot of *FOSL2* and its target genes, with each target gene colored by log fold change between post-induced chondro and pre-induced chondro.

The induction of MSCs to chondrocyte-like cells has important implications for cartilage and bone regeneration (Pittenger et al., 1999). Since UC-MSCs have the highest fraction of chondro, UC-MSCs were induced to chondrocyte-like cells following our previous study (Li et al., 2019). We conducted scRNA-seq on chondrogenesis-induced MSCs and obtained 6,161 high-quality single-cell transcriptomes for further analyses. We conducted an integration analysis of UC-MSCs and chondrogenic-induced MSCs for a better understanding of their relationships (Figure 3A). We found chondrogenic-induced osteo and chondrogenic-induced adipo partially overlapped with their counterparts in pre-induced UC-MSCs on t-distributed Stochastic Neighbor Embedding (t-SNE) plot (Figure 3B). On the other hand, chondrogenic-induced chondro and its pre-induced counterpart are completely separated from each other (Figure 3B), indicating the states of chondro changed a lot after chondrogenesis induction. The expressions of lineage-specific genes, such as *HMGB2*, *HIST1H4C*, *BGN*, *S100A10*, *TXN*, *FKBP1A*, *CXCL2*, *IL6, FGF2, MDK,* and *MMP14*, are essentially consistent pre-and post-induction, although their expressions varied somewhat (Figure 3C, Supplementary Figure S3A). Chondrogenic-induced chondro has a significantly higher chondrocyte score compared to its pre-induced counterpart (Figure 3D, Supplementary Figure S3B), indicating the chondrogenesis induction works well. The fraction of chondro in chondrogenic-induced MSCs (91%) is much higher than its counterpart in pre-induced MSCs (67%) (Figure 3E), indicating chondro expansion during the induction. On the other hand, the fractions of oseto (1.6%) and adipo (7.7%) in chondrogenic-induced MSCs were much lower than that of their counterparts in pre-induced MSCs (Figure 3E), thus both oseto and adipo relatively shrank, potentially due to the induction being unfavorable for their proliferation. Entropies of chondrogenic-induced MSC subpopulations were significantly lower than their counterparts in pre-induced MSCs (Supplementary Figure S3C), implying the decreased stemness during induction. These results indicated both dynamics of cell subpopulation and changes of cell status played roles during chondrogenesis induction.

We identified 764 differentially expressed genes between chondrogenic-induced chondro and its pre-induced counterpart. The induced chondro-specific genes are enriched in positive regulation of cartilage development (4.6 × 10^−4^), HIF-1 signaling pathway (7.9 × 10^−6^), PPAR signaling (4.5 × 10^−3^), and so on (Figure 3F). The induced chondro-specific genes include *COL6A1*, *COL6A2*, *DCN*, *FGF2*, and *MMP14* (Figure 3G and Supplementary Table S2), which play important roles in collagen formation or priming chondrogenic progenitors (Zelenski et al., 2015). Furthermore, the enrichment of the HIF-1 signaling pathway is consistent with previous reports that low oxygen levels could promote chondrogenic differentiation (Krinner et al., 2009). The cell cycle-related genes (*BIRC5*, *CCND1*, *PCNA*, *TPX2*, *AURKA*) are enriched in pre-induced chondro specific genes, consistent with the aforementioned reduced stemness and more differentiated states. On the other hand, the osteoblast lineage-specific genes (*COL1A1*, *COL1A2, SPARC*, *TGFBI*) and adipocyte/myocyte lineage specific genes (*FKBP1A*, *TAGLN*) have been down-regulated in chondrogenic-induced osteo and chondrogenic-induced adipo, respectively (Supplementary Figures S3D,E and Supplementary Table S2). These observations indicate chondrogenesis induction promotes chondro differentiation while represses adipo differentiation and oseto differentiation, reflecting the heterogeneous response of MSCs to induction.

The cell-cell crosstalk of chondro-chondro significantly increase after chondrogensis induction (Figure 3H), which may indicate the crosstalk between chondros played an important role during chondrogensis induction. In order to better understand the processes and mechanisms during chondrogenesis induction, we identified induction-associated transcription factors (TFs) networks. The top up-regulated TFs networks are *FOSL2, ATF5, FOXF1, HES7*, and so on, among which *FOSL2* regulated many target genes including *LDHA*, *SAA1*, *BNIP3*, *COL6A1*, *CDKN1A*, and *FGF2* (Figure 3I) (Karreth et al., 2004), potentially indicating *FOSL2* plays a key role in chondrogenesis induction.

### Heterogeneous Response of MSCs to Osteogenesis Induction

**FIGURE 4:**
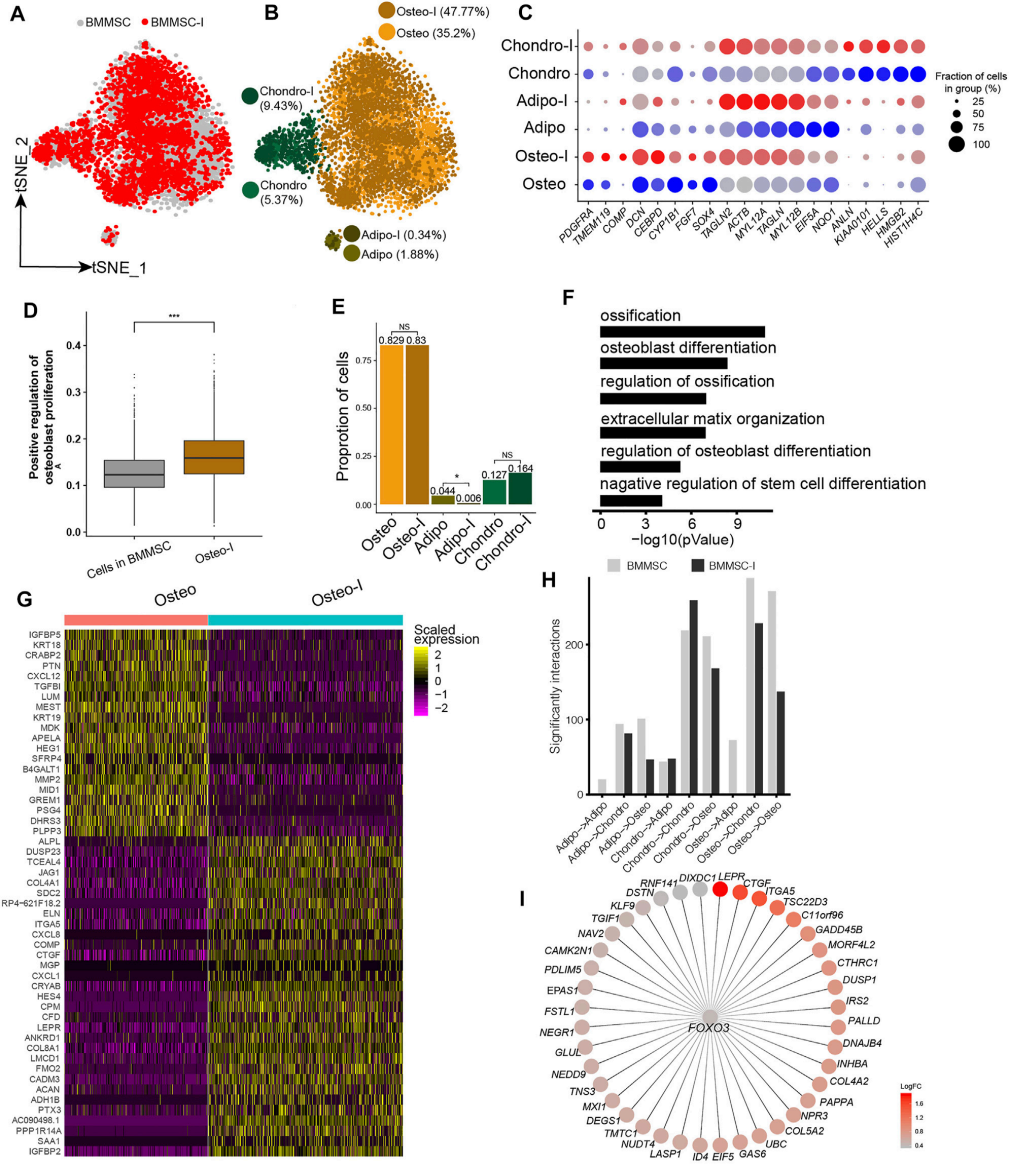
Response heterogeneity and dynamics of MSCs to osteogenesis induction and its underlying mechanisms. **(A)**. t-SNE projection of MSCs pre-and post-osteogenesis induction, colored by non-induced BM-MSC (BM-MSCs) and osteogenic-induced MSCs (BMMSC-I). **(B)**. t-SNE projection of MSCs pre-and post-osteogenesis induction, colored by the six MSC subpopulations, namely chondro, osteo, adipo, chondro-I, osteo-I, and adipo-I. **(C)**. Dot plot of the expression level of lineage-specific genes in MSC subpopulations pre- and post-osteogenesis induction. **(D)**. Boxplot of osteocyte differentiation scores of cells in BMMSC and post- induced osteo (osteo-I). Wilcoxon Rank Sum test, ***p < 0.001. **(E)**. Cell fractions of MSC subpopulation in BMMSCs and BMMSC-I. Fisher’s exact test, *p-value < 0.05. **(F)**. The significantly enriched GO terms in osteo-I specific genes compared to non-induced osteo. **(G)**. Heatmap of differentially expressed genes between pre- and post-induced osteo. **(H)**. Bar plot of significant interaction between MSC subpopulations pairs in BM-MSCs and BMMSC-I. **(I)**. *FOXO3* and its target genes, with each target gene colored by log fold change between osteogenic-induced osteo and pre-induced osteo.

Our analyses showed BM-MSCs have the highest fractions of osteo, and osteo in BM-MSCs showed the highest differentiation potential to osteoblast among all MSC samples. Therefore, BM-MSC is the best candidate among all MSCs for osteogenesis induction. We obtained the scRNA-seq data of BM-MSCs and osteogenic-induced MSCs in Rauch et al. (2019). The BM-MSCs and osteogenic-induced MSCs were essentially overlapped on t-SNE plot (Figure 4A). The MSCs were clustered into oseto, chondro, and adipo (Figure 4B), as aforementioned. The expressions of lineage-specific genes, such as *HMGB2*, *PDGFRA, TMEM119*, *HIST1H4C*, *ACTB, TAGLN, IL6, FGF2,* and *MMP14*, are essentially consistent pre- and post-induction, although their expression varied somewhat (Figure 4C). The osteogenic-induced osteo exhibited increased osteoblast proliferation than pre-induced osteo (Figure 4D), indicating the osteogenesis induction works well. The fractions of osteo and chondro increased little in osteogenic-induced MSCs, while the fraction of adipo significantly decreased (Figure 4E), consistent with the report that osteogensis and chondrogensis share part of the development trajectory while osteogensis and adipogensis are opposite processes (Wolock et al., 2019a; Rauch et al., 2019). The osteogenic-induced MSC subpopulations had lower entropies than their counterparts in pre-induced MSCs (Supplementary Figure S4A), consistent with the observation that differentiated cells have lower entropies/stemnesses.

We identified 2,135 differentially expressed genes between pre-induced osteo and osteogenic-induced osteo. The significantly up-regulated genes in osteogenic-induced osteo enriched in ossification (1.6 × 10^−11^), osteoblast differentiation (3.2 × 10^−9^), extracellular matrix organization (1 × 10^−7^), regulation of osteoblast differentiation (1 × 10^−7^) and negative regulation of stem cell differentiation (6.3 × 10^−5^) (Figure 4F). The most up-regulated genes include *ACAN*, *COL8A1*, *HES4*, *CTGF*, *ITGA5*, *JAG1*, *ALPL*, *TMEM119,* and *COMP* (Figure 4G), among which *ALPL*, *COMP,* and *TMEM119* are well-known osteoblast-specific genes (Supplementary Figure S4B). Both adipo and chondro displayed an increased expression of osteocyte-related genes (*IGFBP2*, *JAG1*, *HES4*), and decreased expression of their lineage-specific gene after osteogenic induction (Supplementary Table S2). Interestingly, the cell-cell crosstalk of osteo-osteo did not show an increase after osteogensis induction (Figure 4H), but strong cell-cell crosstalk in chondro-chondro, potentially indicating the crosstalk between the chondro and osteo play important role in osteogensis induction. We further identified osteogensis associated TF networks such as *MAFF, FOXO1, MXI1*, among which *FOXO3* up-regulated many genes including *LEPR*, *CTGF*, *ITGA5,* and *COL5A2* (Figure 4I), consistent with recent studies (Hamidouche et al., 2009; Ambrogini et al., 2010).

### Heterogeneous Response of MSCs to Adipogenesis Induction

**FIGURE 5:**
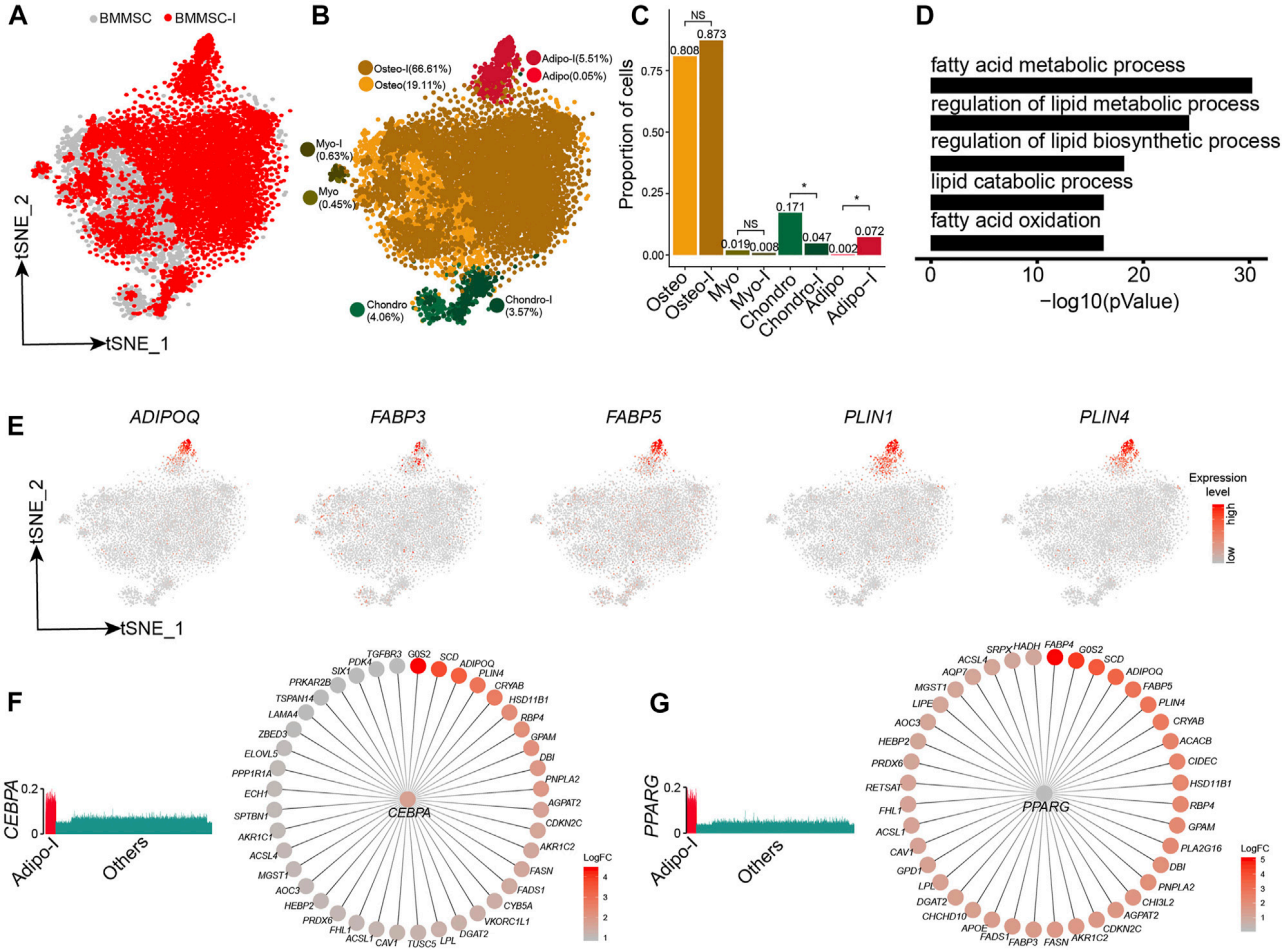
Response heterogeneity and dynamics of MSCs to adipogenesis induction and its underlying mechanisms. **(A)**. t-SNE projection of MSCs pre-and post-adipogenesis induction, colored by non-induced BM-MSC (BM-MSCs) and adipogenic-induced MSCs (BMMSC-I). **(B)**. t-SNE projection of MSCs pre-and post-adipogenesis induction, colored by MSC subpopulations, namely chondro, osteo, adipo, myo, chondro-I, osteo-I, adipo-I, and myo-I. **(C)**. Fractions of MSC subpopulation in BM-MSCs and adipogenesis-induced MSCs. Fisher’s exact test, * *p*-value < 0.05. **(D)**. Significantly enriched GO terms in adipo-specific genes. **(E)**. Expression of adipogenic lineage-specific genes on t-SNE projection of MSCs. **(F)**. Expression of *CEBPA* in single cells **(left panel)**, *CEBPA* and its target genes, with each target gene colored by log fold change between adipogenesis-induced adipo and pre-induced adipo **(right panel)**. **(G)**. Expression of *PPARG* in single cells **(left panel)**, *PPARG* and its target genes, with each target gene colored by log fold change between adipogenesis-induced adipo and pre-induced adipo **(right panel)**.

We further analyzed the responses of BM-MSCs to adipogenesis induction using scRNA-seq data. The BM-MSCs and adipogenic-induced MSCs were mostly overlapped on t-SNE projection (Figure 5A). Unsupervised clustering of BM-MSCs and adipogenic-induced MSCs identified four MSC subpopulations, namely oseto, chondor, adipo, and myoblast (myo) (Figure 5B, Supplementary Figure S5A). Adipo emerged after adipogenesis induction since there is almost no adipo in pre-inducted MSCs, while the fraction of the other three MSC subpopulations either decreased or unchanged (Figures 5A–C). All MSC subpopulations in adipogenic-induced MSCs had lower entropies compared with their counterparts in pre-induced MSCs, indicating a more differentiated cell state after adipogenesis induction (Supplementary Figure S5B).

Adipogenic-induced adipo significantly enriched genes associated with adipocytes, such as fatty acid metabolic process (8 × 10^−31^), regulation of lipid metabolic process (6.3 × 10^−25^), and regulation of lipid biosynthetic process (6.3 × 10^−19^) (Figure 5D, Supplementary Figure S5C). The adipocyte lineage-specific genes, such as *ADIPOQ*, *FABP3*, *FABP5*, *PLIN1,* and *PLIN4* (Hu et al., 1996; Yamamoto et al., 2016), almost exclusively expressed in adipo (Figure 5E). Conversely, osteo, myo, and chondro displayed decreased expression of their lineage-specific gene after adipogenic induction (Supplementary Figures S5D–F). We identified the TF networks, such as *CEBPA, PPARG,* and *STAT5A*, played an important role during adipogenesis induction. *CEBPA* and *PPARG* networks co-regulated a lot adipogenic associated genes such as *ADIPOQ*, *PLIN4*, *FABP4,* and *FABP5* (Figures 5F,G), consistent with previous studies (Rosen et al., 2002; Lefterova et al., 2014). The results indicated that adipogensis induction lead to pronounced transcriptome change, different from the mild change during chondrogensis induction and osteogensis induction.

## Discussion

Here, we analyzed 26,484 high-quality single-cell transcriptomes in eight MSC samples from UC, SY, BM, and AD pre and post lineage-specific induction. By applying unsupervised clustering of single-cell transcriptomes for each sample and across samples, we found similar MSC subpopulations across different tissue sources. We found three MSC subpopulations within each tissue source, namely osteo, adipo, and chondro, which is consistent with the main developmental lineage of MSCs. MSCs from different tissues showed pronounced differences on the abundances of MSC subpopulations, indicating that it is critical to select appropriate tissues to collect MSCs. Although there are many tissues that could be used for the collection of MSCs, we only analyzed the MSCs in four commonly used tissues which could be representative. We noticed that our observations are quite different from a recent report by Huang et al. (2019) showing very limited heterogeneity in UC-MSCs. The observation of limited heterogeneity in Huang et al. could be attributed to the very small number of cells (∼200) in their study.

UC-MSCs are considered as an attractive substitute for BM-MSCs in clinical application because of their simple preparation, strong proliferation ability, and low immunogenicity (Lu et al., 2006). A recent study investigated the differences in gene expression profiles between BM-MSCs and UC-MSCs (Barrett et al., 2019), but missed the comparison among the their subpopulation counterparts. We directly compared the lineage specific differentiation potential between the subpopulation counterparts thanks to consistent MSC subpopulations across tissues. Compared with other MSCs, UC-MSCs clearly showed much stronger immunomodulation capability, which does not show pronounced subpopulation differences, indicating much higher baseline expression of immunomodulation genes including *IL6*. On the other hand, we found differentiation potentials of MSC subpopulations counterparts between BM and UC were quite different. E.g., chondro in BM showed a much higher differentiation score to chondrocyte than chondro in UC, while adipo in UC showed a much higher differentiation score to adipocyte than adipo in BM. Therefore, BM and UC showed differences in differentiation potentials to specialized functional cells thus BM has its own niche in clinical application. Analyses of single-cell RNA-seq data of MSCs before and after osteogenesis induction, chondrogenesis induction, and adipogenesis induction revealed that MSC subpopulations respond to these inductions quite differently. Based on our observation of osteogenesis induction and chondrogenesis induction, the MSC subpopulation is expanded and differentiated when they were consistent with induction directions, while other subpopulations shrunk. Adipogenesis induction lead to the emergence of the adipo, thus is associated significantly with a change of gene expression profiles on this lineage. The observations are consistent with the report by Rauch et al. (2019) that adipogenesis showed much significant transcriptomic and epigenomic changes than that of osteogenesis. By comparing TF regulon activity before and after each lineage-related induction, we identified a set of upregulated TFs in each differentiational lineage. In particular, *FOSL2, FOXO3,* and *CEBPA* regulate many target genes during chondrogenesis, osteogenesis, and adipogenesis respectively, potentially indicating their key roles in MSC induction. These genes could be potential targets for efficient induction to facilitate specific clinical applications.

Overall, we integrated the scRNA-seq data of MSCs in multiple tissues, which provide a unified platform and perspective for the understanding of the heterogeneity of MSCs and their response to different inductions we characterized the MSC subpopulations and their heterogeneous response at single-cell resolution, which facilitated choosing suitable MSCs for clinical usage. Further study of MSCs using multiple single-cell omic technologies as well as target gene knockout assays could provide more detail about each MSC subpopulation and their differentiation mechanisms (Jin et al., 2015; Lai et al., 2018; Baccin et al., 2019; Xu et al., 2021), facilitating the development of MSC-based clinical applications.

## Materials and Methods

### Collection and Culture of Human UC-MSCs

The human UCs were collected from Shenzhen Second People’s Hospital. The healthy donors signed an informed consent form approved by the IRB of Shenzhen Second People’s Hospital. UC-MSCs were isolated from Wharton jelly of umbilical cord and cultured following our previous study (Li et al., 2019). In brief, the UCs were obtained from normal deliveries according to the institutional guidelines. The UCs were immediately put in physiological saline containing heparin anticoagulant at 4°C after collection, which was further processed within 6 h. The UCs were cut into 3–5 cm long segments under a sterile environment. Vessels of umbilical cords were removed, and Wharton’s jelly was collected. The Wharton’s jelly was cut into small pieces (2–3 mm^3^), which were placed in a petri dish with MSCs culture medium (MesenGro ®human MSC Medium, StemRD, US), 10% fetal bovine serum (FBS; Gibco, Australia), and 10 μg/L basic fibroblast growth factor (bFGF; Gibco, Australia). The Wharton’s jelly blocks were cultured at 37°C in a 5% CO_2_ incubator. Fresh medium was added to the flasks after 3 days. Tissue blocks were removed after 7 days in culture and the medium was replaced. Medium replacement was carried out every 72 h until the cells reached an 80% confluent layer. Cells were harvested with 0.25% (w/v) trypsin plus 0.02% (w/v) EDTA (Hyclone, USA) and sub-cultured at a density of 1,000 cells/cm^2^.

### Collection and Culture of Human SY-MSCs

The human synovial tissue was collected from Shenzhen Second People’s Hospital. The healthy donor signed an informed consent approved by the IRB of Shenzhen Second People’s Hospital. SY-MSCs were isolated from the synovial membrane of knee joints of the healthy donor and cultured following De Bari et al. (2001). The synovial membrane was immediately put in physiological saline containing heparin anticoagulant at 4°C after collection, which was further processed within 6 h. Then the synovial membrane was minced and digested with 0.2% collagenase in high-glucose Dulbecco’s modified Eagle’s medium (DMEM; Gibco, Australia) containing 10% fetal bovine serum (FBS; Gibco, Australian) and 10 μg/L basic fibroblast growth factor (bFGF; Gibco, Australian). Following overnight incubation at 37°C with 5% CO_2_, cells were collected by centrifugation, washed twice, resuspended in high-glucose DMEM supplemented with 10% FBS and 10 μg/L bFGF, plated in a culture flask, and allowed to attach for 3 days. Nonadherent cells were removed after 7 days of culture and the medium was replaced. Medium replacement was carried out every 72 h until the cells reached an 80% confluent layer. Cells were harvested with 0.25% (w/v) trypsin plus 0.02% (w/v) EDTA (Hyclone, USA) and sub-cultured at a density of 1,000 cells/cm^2^.

### Chondrogenesis Induction

Chondrogenesis induction was conducted following our previous study (Li et al., 2016). In brief, UC-MSC was induced to chondrocyte-like cells by chondrocytes specific medium. In monolayer culture, UC-MSCs were supplemented with 0.1 mM dexamethasone, 40 mg/ml L-proline, 10 μg/L transforming growth factor beta-1 (TGF-β1, Peprotech, USA), 10 μg/L insulin-like growth factor-1 (IGF-1) (Peprotech, USA), and 1% insulin transferrin selenium (ITS, Invitrogen). The cells were incubated for 3 weeks at 37°C in a humidified atmosphere of 5% CO_2_ and the medium changed every 3 days.

### scRNA-Seq

The UC-MSC and chondrogenic-induced MSC were obtained directly from the cultured cells. FACS sorting was performed on a Becton Dickinson FACSAria II (BD Biosciences, Denmark) to remove the dead cells. scRNA-seq was conducted using 10X genomics platform. Chromium Single Cell 3′Gel Bead and Library Kit (P/N 120237, 120236, 120262, 10x Genomics) were used following protocols. Approximately 15,000 cells were loaded per channel. Sequencing libraries were loaded on Illumina NovaSeq 6000 with paired-end kits.

### Pre-Processing of scRNA-Seq Data

Raw sequencing data were converted to FASTQ format with demultiplexing using Illumina bcl2fastq. Cell Ranger Single-Cell Software Suite (V2.2.0 10X Genomics; https://support.10xgenomics.com) was used to perform reads alignment, barcode demultiplexing. The reads were aligned to the hg38 reference genome. Digital gene expression matrices were preprocessed and filtered using R packages scran and scater (Lun et al., 2016). Cells with more than 4,000 expressed genes (potential doublets), less than 500 expressed genes (potential low-quality libraries), and more than 10% of mitochondrial UMI counts (potential cell fragments and debris) were filtered out. Additionally, we applied Scrublet (Wolock et al., 2019b) to identify potential doublets. The doublet score for each single cell and the threshold based on the bimodal distribution was calculated by default parameters. The expected doublet rate was set to be 0.08, and cells predicted to be doublets or with doubletScore larger than 0.25 were filtered out. A total of 26,484 cells were left after quality control. Normalization of UMI count was performed by first dividing UMI counts by the total UMI counts in each cell, followed by multiplication with the median of the total UMI counts across cells.

### Dimension Reduction and Visualization of scRNA-Seq Data

Seurat (Butler et al., 2018) is used for data integration, data normalization, dimension reduction, cell clustering, and other basic scRNA-seq data analyses following our previous studies (Zhou and Jin, 2020; Qin et al., 2021). To avoid highly expressed genes dominating later analyses, we scaled the mean and variance of each gene across cells to 0 and 1, respectively. The scaled expression data was used for the selection of highly variable genes that were used for conducting dimension reduction. UMAP (Becht et al., 2018) or t-SNE (van der Maaten and Hinton, 2008) is used for the visualization of scRNA-seq data. The cells were clustered using unsupervised graph clustering in SNN-Cliq (Xu and Su, 2015) and PhenoGraph (Levine et al., 2015).

### Identification of Cluster-Specific Genes and Differentially Expressed Genes

Gene expressions of each investigated cluster were compared to these of the remaining clusters by Wilcoxon Rank Sum test. The genes that are significantly highly expressed in the investigated cluster were regarded as cluster-specific genes. The significantly differentially expressed genes between two clusters were also tested by Wilcox Rank Sum test. A cut-off of minimum log2(fold change) of 0.25 and adjusted *p*-value of 0.01 were used to determine the significantly differentially expressed genes. We used Metascape (http://metascape.org) (Zhou et al., 2019) to perform biological process enrichment analysis.

### Specific Functional Program and Cell Scoring

We calculated the cell score of each cell to represent its differentiation potential to a specific functional cell, similar to Tirosh et al. (2016). Briefly, functional cells such as osteocyte, chondrocyte, and adipocyte have specifically expressed genes that are defined as functional-program, and their average relative expression as the functional-program cell score. For a given gene *i* in functional-program, its expression in cell *j* was scored S_ij_, thus the cell score S = ∑∑S_ij_/i*j. To decrease the effect of the data quality of the cell on functional-program score, we defined control gene-sets and their average relative expression as control scores. The analyzed gene set was binned into 30 bins of equal size. We randomly chose 100 genes from the expression bin of each gene set as the control gene-set. We calculated the Z-score of the gene expression as the final gene set score.

### MetaNeighbor Analysis

To compare the similarities among MSC subpopulation counterparts from different tissue sources, we performed MetaNeighbor (Crow et al., 2018) analysis using the R function “run_MetaNeighbor” to assess the similarity of cluster counterparts in different tissues.

### Inferring Single-Cell Regulatory Network by SCENIC

Based on the raw count matrices and following the proposed workflow, activated regulons in each MSC subpopulation were inferred using SCENIC (Aibar et al., 2017) using the default parameters. The differentially activated regulons in each MSC subpopulation were identified by the Wilcoxon Rank Sum test compared with the control MSC subpopulation.

### Ligand-Receptor Interaction Analysis

To quantify the interaction between each pair of MSC subpopulations, we adopted a network-based method (Farbehi et al., 2019) to build a weighted cell-cell interaction network, where the edge weight is determined by log2 fold-change of each ligand and receptor in source and target cells as well as protein-protein interaction probability based on STRING database.

### Single-Cell Signature Exploration

We applied Single Cell Signature Explorer (Pont et al., 2019) to quantify geneset-based signature at single-cell level with default settings. The GO terms were downloaded from MSigDB v6.2 (http://www.broad.mit.edu/gsea/msigdb/index.jsp).

### Single-Cell Entropy

We applied a modified version of single-cell entropy calculation from Teschendorff and Enver (2017), which were shown as:

$$E_{n}=-\sum_{m=1}^{m=M}\frac{p_{m,n}*\log\left(p_{m,n}+10^{-10}\right)}{\log\left(M\right)},$$

Where $E_{n}$ represents the entropy of a cell n. For a population of N cells with M genes, we can then define a probability distribution $p_{m,n}={\text{UMI}}_{m,n}/\sum_{m=1}^{m=M}{\text{UMI}}_{m,n}$ as the probability of gene m expressed in cell *n*.

### Statistical Analysis

All statistical analyses and graphics were conducted using R. The Wilcoxon Rank Sum test was performed to identify the significantly differentially expressed genes between two cell clusters. Bonferroni correction was conducted for multiple testing corrections.

## Data Availability

The raw sequence data reported in this paper have been deposited in the Genome Sequence Archive in BIG Data Center, under accession numbers HRA000086. The publicly available datasets: 1) MSCs derived from adipocyte (SRP148833); 2) bone marrow-derived MSCs, adipogenesis-induced BMMSC and osteogenesis-induced BMMSC (GSE113253).
