# Supplementary material for: Cross-Tissue Characterization of Heterogeneities of Mesenchymal Stem Cells and Their Differentiation Potentials
Source: Front Cell Dev Biol. 2021 Dec 17;9:781021. doi: 10.3389/fcell.2021.781021 (PMC8719164; doi:10.3389/fcell.2021.781021)
Supplement: Supplementary file 2 [file DataSheet1.docx]

**Figures**

**
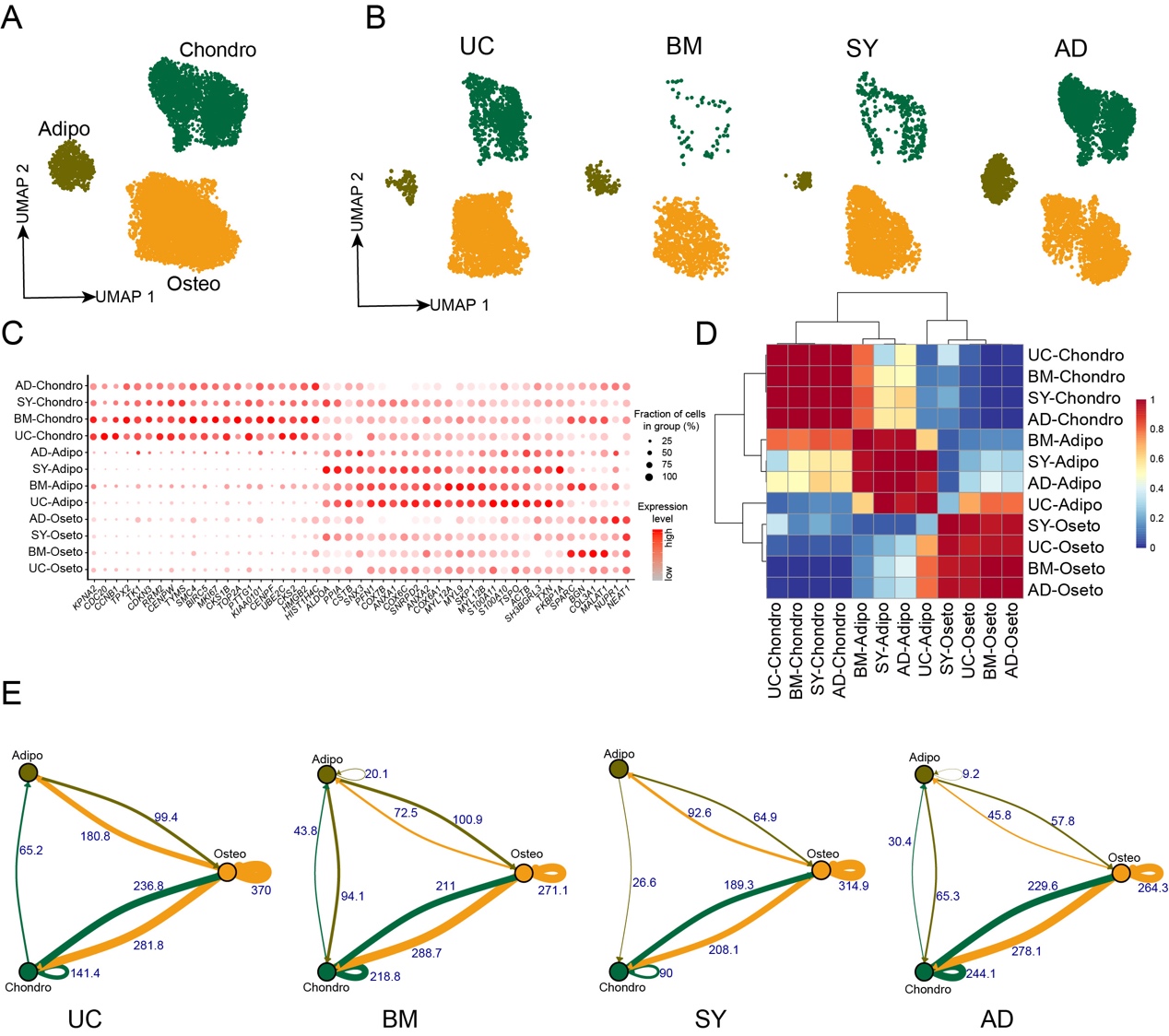
**

**Figure 1. MSC subpopulations in 4 different tissues.**

**
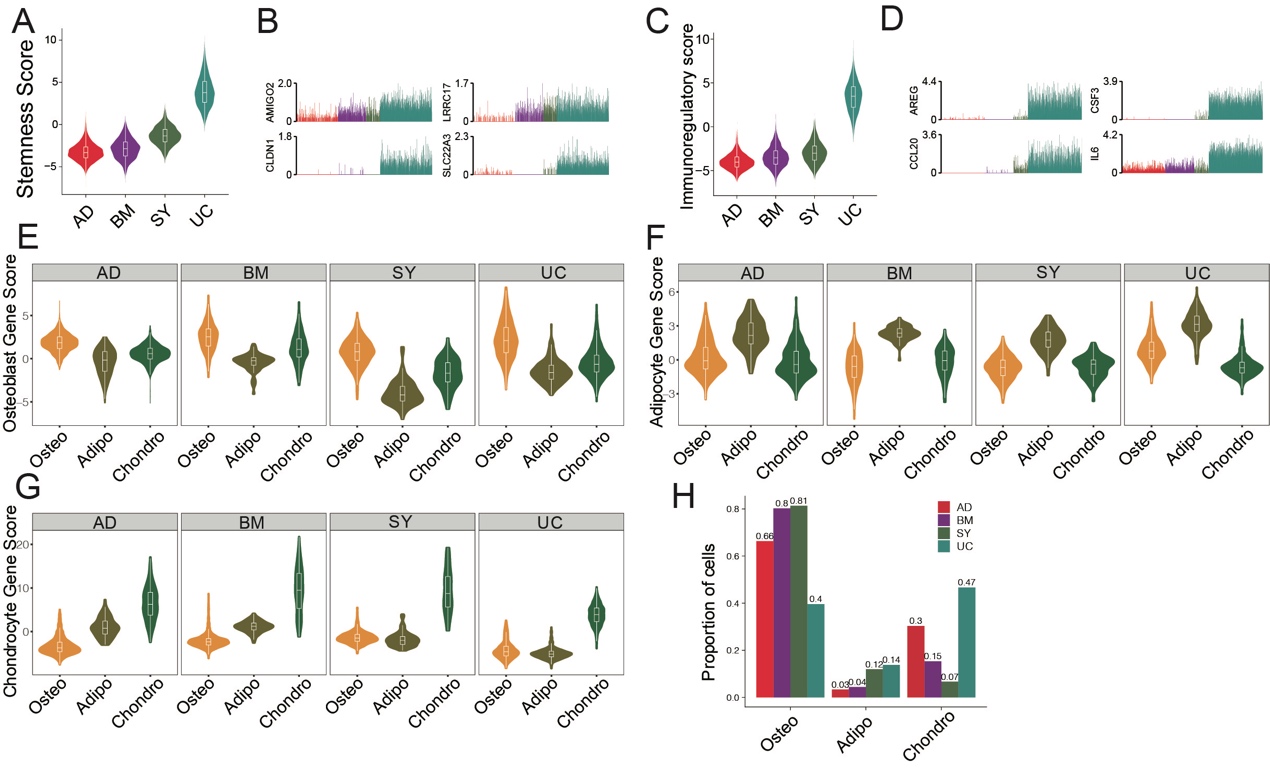
**

**Figure 2. Features and lineage specific differentiation potentials of MSC subpopulations.**

**
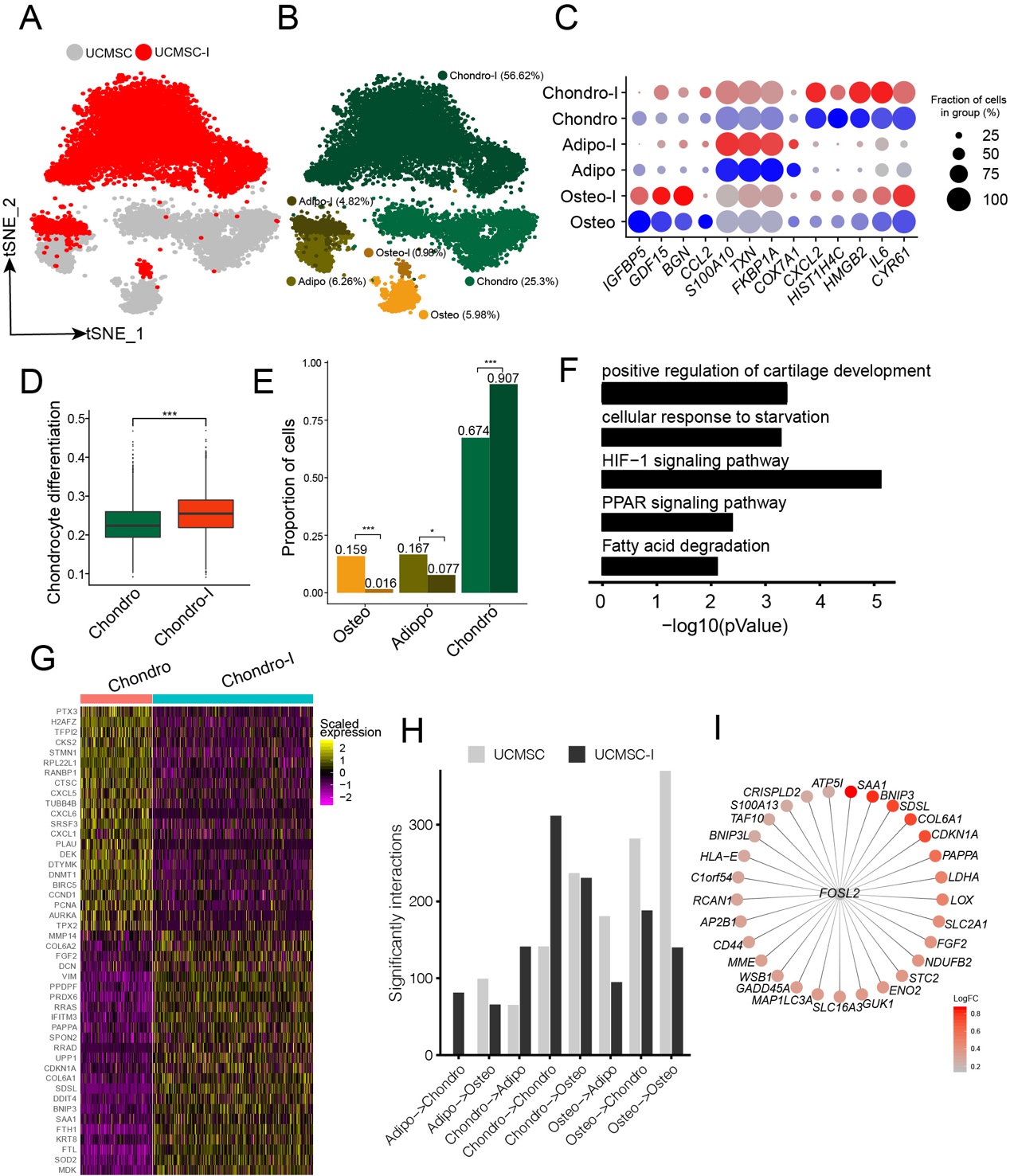
**

**Figure 3. Response heterogeneity and dynamics of MSCs to chondrogenesis induction and its underlying mechanisms.**


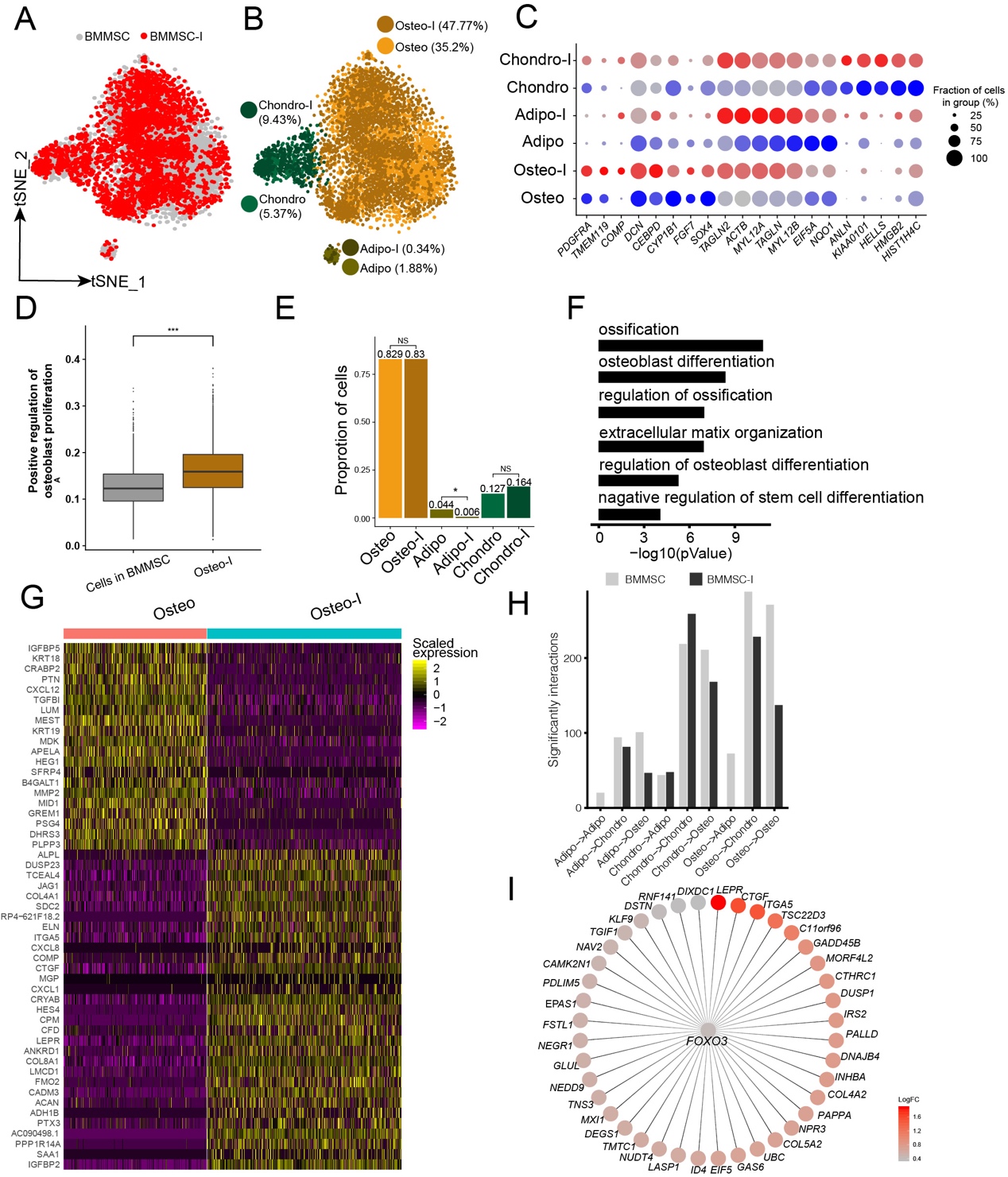


**Figure 4. Response heterogeneity and dynamics of MSCs to osteogenesis induction and its underlying mechanisms.**

**
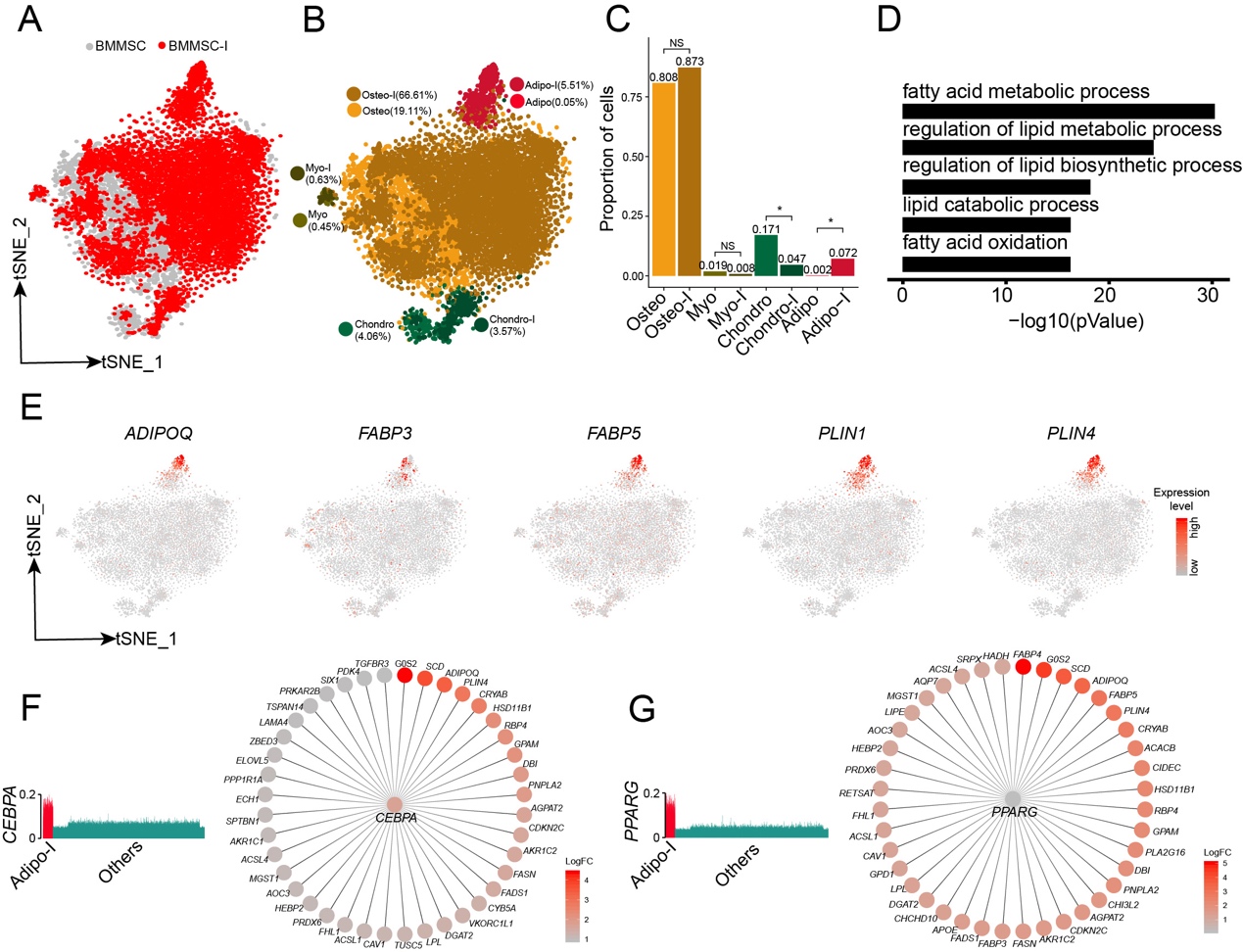
**

**Figure 5. Response heterogeneity and dynamics of MSCs to adipogenesis induction and its underlying mechanisms.**


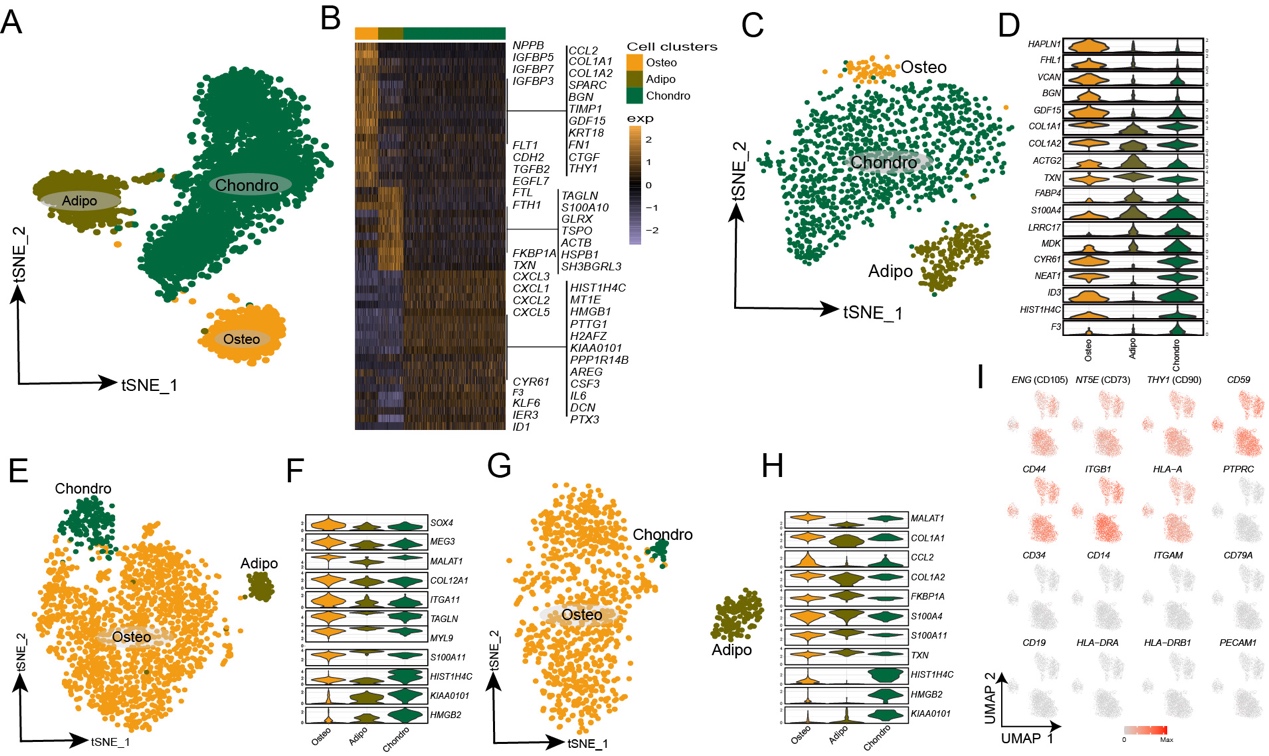


**Figure S1. The MSC subpopulations in each MSC sample.**

**
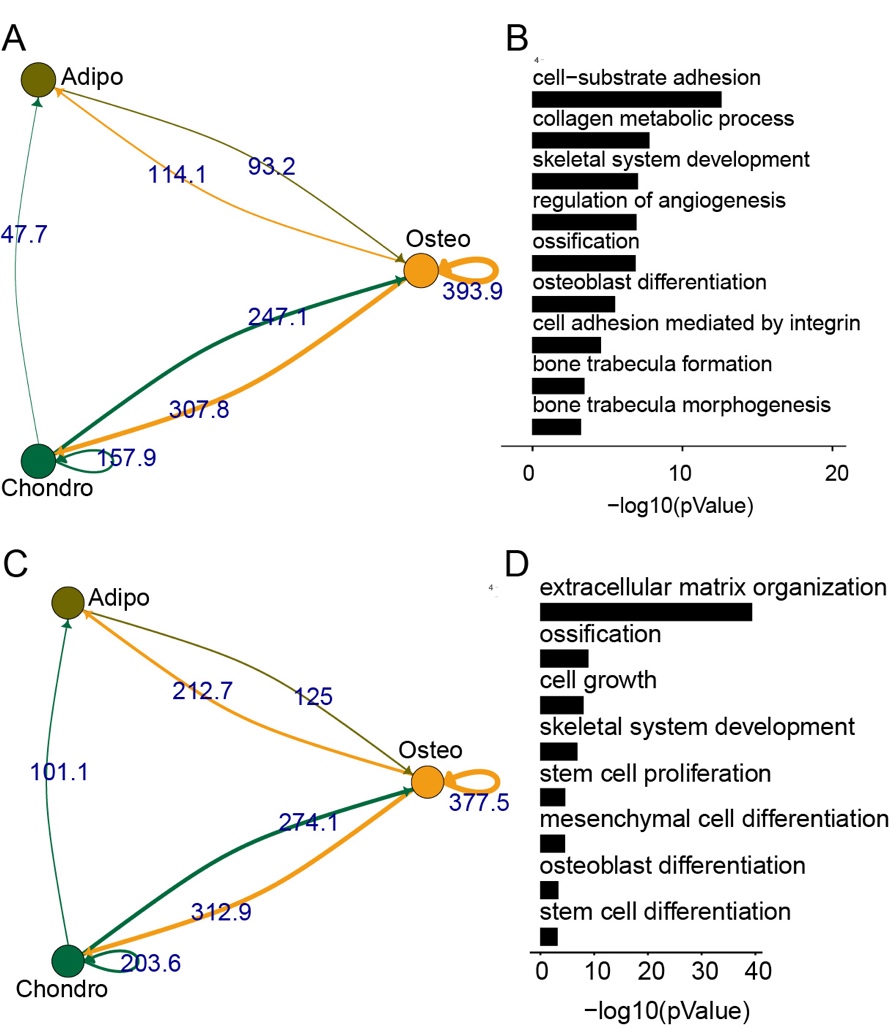
**

**Figure S2.** **Cell-cell crosstalk between MSC subpopulations based on ligand-receptor pairs.**

**
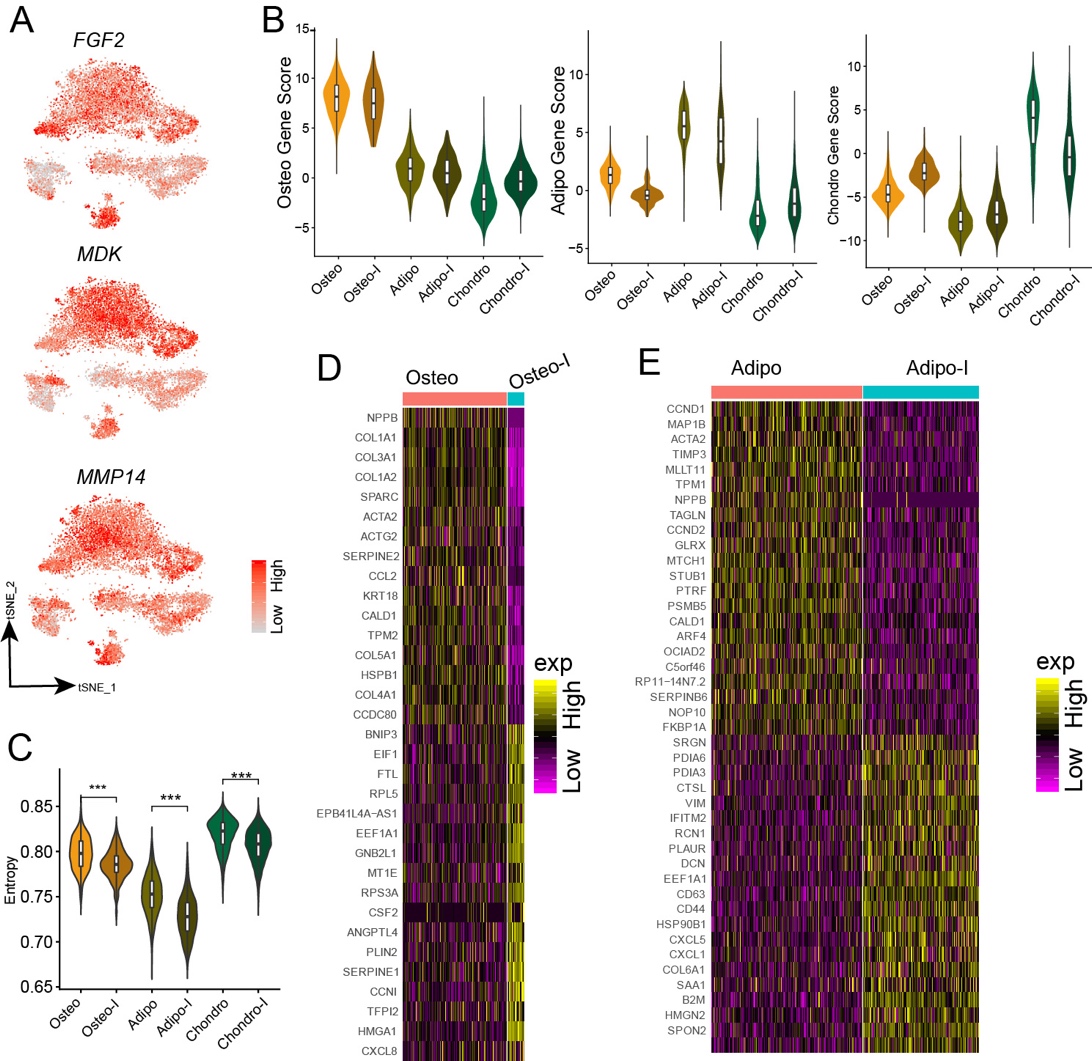
**

**Figure S3. Characteristics of MSC subpopulations and their response to chondrogenesis induction in UC-MSC.**


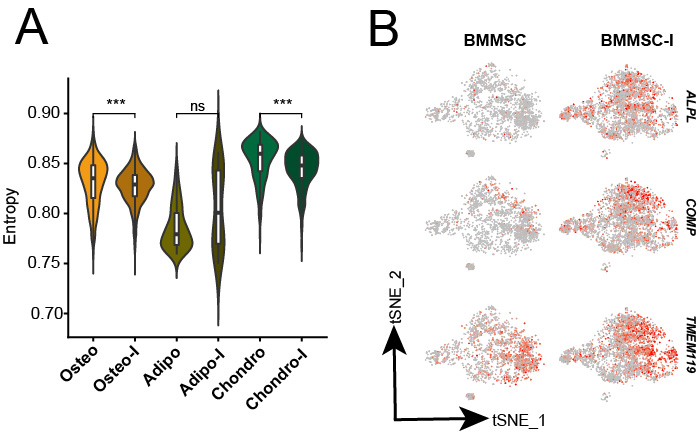


**Figure S4. Characteristics of MSC subpopulations and their response to osteogenesis induction in BM-MSC.**


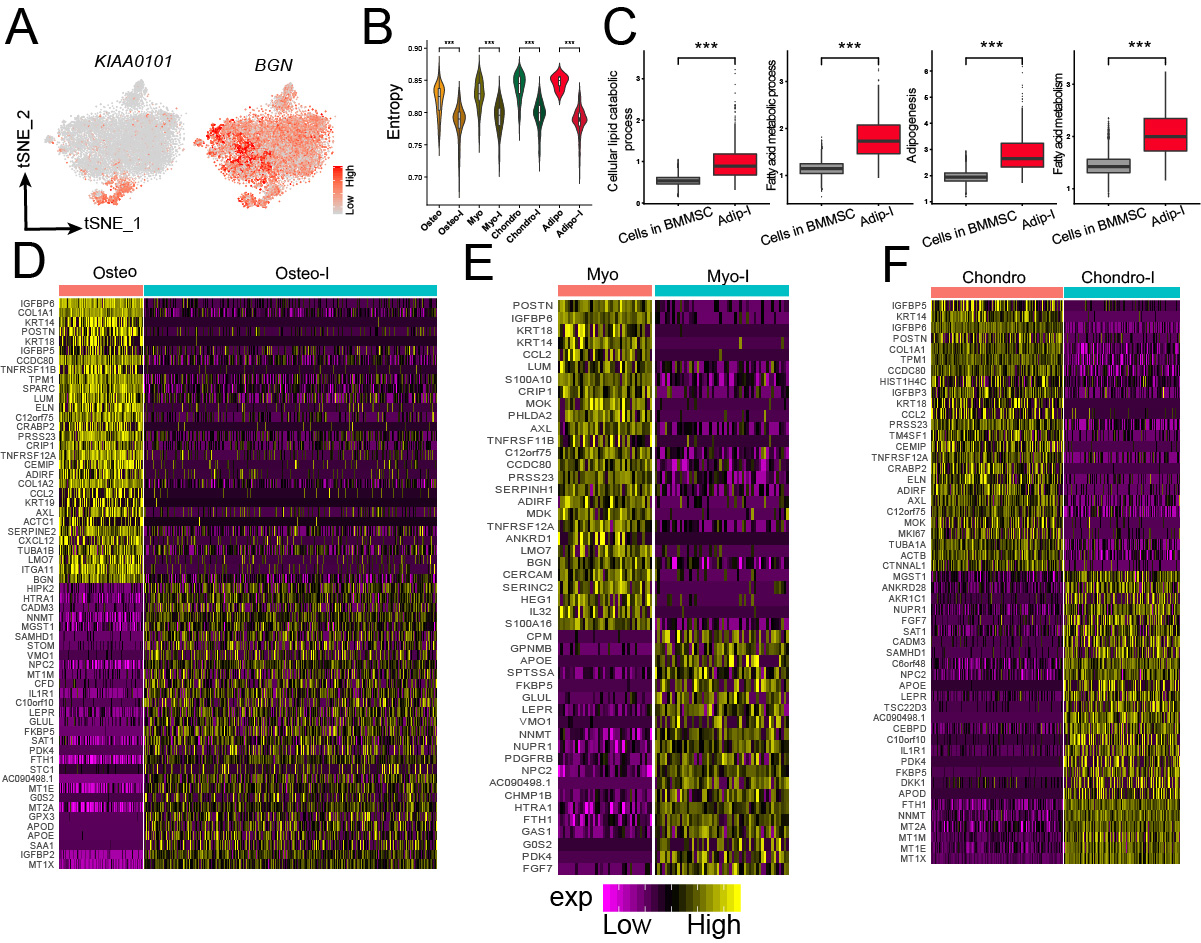


**Figure S5. MSC subpopulations’ features and their response to adipogenesis induction****.**
